# Obesity should not be considered a contraindication to medial Oxford UKA: long-term patient-reported outcomes and implant survival in 1000 knees

**DOI:** 10.1007/s00167-018-5218-6

**Published:** 2018-10-25

**Authors:** James Molloy, James Kennedy, Cathy Jenkins, Stephen Mellon, Christopher Dodd, David Murray

**Affiliations:** 1 Nuffield Department of Orthopaedics, Rheumatology and Musculoskeletal Sciences, University of Oxford, Windmill Road, Oxford, OX3 7LD UK; 2 Nuffield Orthopaedic Centre, Oxford University Hospitals NHS Foundation Trust, Oxford, UK

**Keywords:** Unicompartmental knee arthroplasty, Body mass index, Patient-reported outcome, Implant survival

## Abstract

**Purpose:**

Some health providers ration knee arthroplasty on the basis of body mass index (BMI). There is no long-term data on the outcome of medial mobile-bearing unicompartmental knee arthroplasty (UKA) in different BMI groups. This study aimed to determine the effect of patient body mass index (BMI) on patient-reported outcomes and long-term survival of medial UKA in a large non-registry cohort. Our hypothesis is that increasing BMI would be associated with worse outcomes.

**Methods:**

Data were analysed from a prospective cohort of 1000 consecutive medial mobile-bearing Oxford UKA with mean 10-year follow-up. Patients were grouped: BMI < 25, BMI 25 to < 30, BMI 30 to < 35 and BMI 35+. Oxford Knee Score (OKS) and Tegner Activity Score were assessed at 1, 5 and 10 years. Kaplan–Meier survivorship was calculated and compared between BMI groups.

**Results:**

All groups had significant improvement in OKS and Tegner scores. BMI 35 + kg/m^2^ experienced the greatest overall increase in mean OKS of 17.3 points (*p* = 0.02). There was no significant difference in ten-year survival, which was, from lowest BMI group to highest 92%, 95%, 94% and 93%.

**Conclusion:**

There was no difference in implant survival between groups, and although there was no consistent trend in postoperative OKS, the BMI 35+ group benefited the most from UKA. Therefore, when UKA is used for appropriate indications, high BMI should not be considered to be a contraindication. Furthermore rationing based on BMI seems unjustified, particularly when the commonest threshold (BMI 35) is used.

**Level of evidence:**

III.

## Introduction

Obesity is a well-documented risk factor for the development of knee osteoarthritis (OA) [2], and rising levels of global obesity are predicted to increase demand for knee arthroplasty surgery [8, 19]. However, obesity is widely considered to be a contraindication to knee arthroplasty surgery due, in part, to concern over reduced long-term implant survival. Recently, arthroplasty services in the United Kingdom have been rationed based on body mass index (BMI) [29].

Unicompartmental and total knee arthroplasties (UKA and TKA) are treatment options for end-stage medial compartment OA. Many studies indicate that compared to TKA, UKA provides superior function, faster recovery, lower costs, more normal knee kinematics, and less morbidity and mortality, but the revision rate tends to be higher [20, 25, 28, 32]. However, the revision rate of UKA varies considerably, primarily because surgeons use different indications [15, 21].

Patient weight and BMI increase the risk of revision surgery for TKA [17]. Variable results have been reported for fixed-bearing UKA [4–6, 30, 33, 35], but increased risk with BMI has not been reported for Oxford UKA [18, 23, 27]. However, previous studies investigating the effect of BMI on the outcome of UKAs have been limited by small cohorts [4, 5, 30, 35], few revisions [4–6, 33, 35], short-term follow-up [4, 5, 18, 24, 35] or binary analyses [4–6, 18, 27].

This study aimed to determine the effect of patient BMI, subdivided into multiple groups, on patient-reported outcomes and on 10-year implant survival following medial Oxford UKA used for the recommended indications. The study hypothesis was that with increasing BMI at surgery, worse outcomes would be reported compared to those with normal range BMI.

## Materials and methods

A prospective cohort of cemented phase 3 medial mobile-bearing Oxford UKAs implanted for the recommended indications [13, 14] was used to assess the influence of BMI on clinical outcomes and implant survival. This cohort has previously been reported [26, 27]. This study was specifically designed to investigate the effect of BMI on the patient-reported outcome and 10-year implant survival of medial Oxford UKA.

Between 1998 and 2009, 1000 consecutive cemented medial mobile-bearing Oxford UKA (Oxford Medial UKA, Phase 3, Zimmer Biomet, Bridgend, United Kingdom) were implanted in 818 patients and prospectively followed up. The recommended evidence-based indications were used for patient selection [13, 14]. BMI did not influence treatment choice. All patients had failed non-operative management. Indications were bone-on-bone medial compartment osteoarthritis, normal joint space width in the lateral compartment, and functionally intact ligaments (in particular anterior cruciate and medial collateral). The state of the patellofemoral joint was ignored unless there was bone loss with grooving of the lateral side. Patient characteristics such as age, weight, activity level, and limb alignment were ignored [27]. These indications can be reliably determined on preoperative radiographs, including stress varus/valgus stress radiographs if appropriate [14], and confirmed by visual inspection at time of surgery. In addition, four cases were performed for medial spontaneous osteonecrosis of the knee. At the time of recruitment, our institution was not subject to BMI restrictions and there was no BMI threshold for referral or surgery. All operations were performed by two designer surgeons (CAFD, DM) using the recommended, minimally invasive technique. Patients underwent a standard postoperative rehabilitation programme. Surgeon UKA usage (the percentage of primary knee arthroplasty practice that is UKA) is ≥ 50%.

Preoperative demographic data including patient age, sex, weight and height were recorded at the time of surgery, and postoperative functional scores were collected and recorded on a prospectively maintained database by independent physiotherapists. Patients were assessed at 1, 5 and 10 years.

Inclusion criteria for this study included a recorded height and weight allowing calculation of BMI, and recorded postoperative patient-reported outcome. There were no exclusion criteria.

### Exposure

Patients were classified into four sub-groups a priori based on BMI at the time of surgery. BMI was calculated as weight divided by the square of the height. Groups were BMI < 25 kg/m^2^ (normal), BMI 25 – <30 kg/m^2^ (overweight), BMI 30 – <35 kg/m^2^ (obese class I) and BMI ≥ 35 kg/m^2^ (obese class II), and are consistent with the classifications of obesity defined by the World Health Organisation [34].

### Outcomes

Clinical and functional outcomes were assessed using the Oxford Knee Score (OKS; 0–48, with 48 the best outcome) [10], and the Tegner activity score (0–10; with 10 being participation as an elite athlete) [31]. Revision was defined as the removal, exchange or addition of any implant component, this includes bearing exchange for bearing dislocation, addition of a lateral UKA for lateral compartment progression, or conversion to TKA.

Ethical approval was sought from the local research ethics committee with formal approval deemed unnecessary under National Health Service research governance arrangements.

### Statistical analyses

One-way analysis of variance (ANOVA) or chi-squared tests were used to determine significant baseline differences. Mean postoperative OKS and Tegner scores were calculated at 1, 5 and 10 years, and tested with ANOVA and Kruskal–Wallis tests between BMI groups. Paired *t* tests or Wilcoxon signed rank tests were used to test differences within BMI groups over time. Revision was quantified with Kaplan–Meier survival analysis [16] and significance tested with a log rank test [1]. Statistical significance was defined as *p* value < 0.05.

### Missing values

Four knees (three patients) could not be traced for follow-up so their revision status remains unknown. Height and/or weight was missing for 44 knees (4%), and postoperative OKS and Tegner scores were missing in 19 knees (2%).

Statistical analyses were performed using R (R Core Team 2016, Vienna, Austria). A power analysis was performed using the minimal clinically important difference reported for OKS [7]. Using the Altman nomogram for a power of 80% at a significance level of 0.05 and using a standard deviation of eight, a sample size of 80 patients was required to detect a clinically important difference between groups. With unequal group sizes, 20 knees in the smallest cohort were required for the study to have adequate power.

## Results

Meeting the inclusion criteria were 956 UKAs (96% of cohort) in 785 patients. The mean follow-up was 10.2 years (SD 3, range 5–16 years) with 51% having greater than 10-year follow-up. The mean age was 67 years (SD 9.6, range 33–87 years; Table 1); increasing BMI categorisation was associated with younger age at surgery (*p* < 0.001). Forty-nine percent were female and there were more females in the BMI categories of < 25 and > 35 kg/m^2^ (*p* < 0.001). Preoperative OKS scores were lower in those with higher BMI (*p* = 0.001).

**Table 1:** Cohort demographics

BMI group	*N* knees	*N* patients	Mean age (SD)	Mean BMI (SD)	Sex (F:M)
< 25	207	202	70.3 (10)	22.6 (3)	0.64
25– <30	433	427	66.4 (10)	27.3 (1)	0.41
30– <35	220	218	64.9 (9)	32.1 (1)	0.45
35+	96	94	61.7 (8)	39.0 (4)	0.56
Entire cohort	956	941	66.59	28.54	0.49
*p* value	N/A	N/A	< 0.001	< 0.001	< 0.001

*BMI* body mass index, *N* number, *N/A* not applicable, *SD* standard deviation

**Fig. 1:**
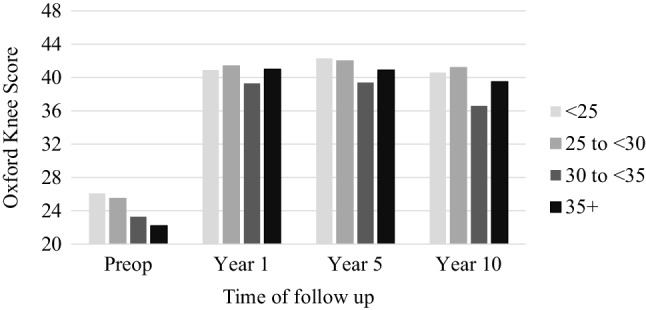
Oxford Knee Score by time of follow-up and BMI group

There was significant improvement between preoperative and postoperative OKS and Tegner scores at 1-, 5- and 10-year follow-up for all groups (Fig. 1; Table 2; *p* < 0.001). Functional scores were lower preoperatively for the heaviest BMI group; however, this group experienced the largest absolute increase in reported OKS over this time period (mean OKS improvement 17.3, Table 2). At 1-, 5- and 10-year follow-up there was a significant difference between the mean OKS and Tegner scores for all groups, except for the Tegner score at 10 years.

**Table 2:** Patient-reported outcomes by BMI classification

BMI	Score	Pre-op	Year 1	Year 5	Year 10	Difference between 10 years and pre-op
< 25	OKS	26.1 (10)	**40.9** (8)	**42.3** (7)	**40.6** (8)	14.5
	Tegner Score	2 [0–6]	**3** [1–6]	3 [0–8]	2 [1–7]	0
25–30	OKS	25.5 (9)	**41.4** (7)	**42.0** (7)	**41.2** (8)	15.7
	Tegner Score	2 [0–8]	**3** [0–7]	**3** [0–10]	3 [0–7]	1
30–35	OKS	23.3 (8)	**39.3** (8)	**39.4** (8)	**36.6** (11)	13.3
	Tegner Score	2 [0–8]	**3** [0–7]	**3** [0–7]	2 [0–6]	0
> 35	OKS	22.2 (9.0)	**41.0** (7)	**40.9** (8)	**39.5** (8)	17.3
	Tegner Score	2 [0–3]	**3** [1–5]	**3** [0–5]	2 [1–5]	0
*p* value between BMI groups for OKS (Kruskal–Wallis Test)		0.001	< 0.001	0.001	0.001	0.02
*p* value between BMI groups for Tegner Score (Kruskal–Wallis Test)		(n.s.)	< 0.001	0.008	(n.s.)	(n.s.)

Mean OKS (SD), Median Tegner [range]

*BMI* body mass index, *OKS* Oxford Knee Score, *ns* not significant

Bold typeface indicates significant difference with pre-op score (p < 0.05)

There were 52 revisions; 26 due to disease progression, 7 for unexplained pain, 7 had bearing dislocation, 6 for infection, 2 for aseptic loosening, and 1 each for instability, malposition and ACL injury. One cause of revision was unknown. 13 of these revisions occurred in the BMI < 25 kg/m^2^ group and represented 6.3% of UKAs in this group. There were 18 (4.2% of group) in the BMI 25– <30 kg/m^2^ group, 10 (4.5%) in the BMI 30 – <35 kg/m^2^ group, and 6 (6.3%) in the BMI ≥ 35 kg/m^2^ group. There was no statistical significance between indications for revision and BMI group (Table 3).

**Table 3:** Component time incidence rate (per 100 years) by revision indication stratified by BMI (95% CI)

BMI	Overall	Lateral OA	Bearing Dis.	Pain	Infection	Malposition	Aseptic loosening	Instability	Other^a^
< 25	0.70 (0.4–1.2)	0.38 (0.2–0.8)	0.05 (0.0–0.4)	0.05 (0.0–0.4)	0.11 (0.0–0.4)	0	0.01 (0.0–0.4)	0.05 (0.0–0.4)	0
25–<30	0.47 (0.3–0.7)	0.20 (0.1–0.4)	0.05 (0.0–0.2)	0.05 (0.0–0.2)	0.10 (0.0–0.3)	0.02 (0.0–0.2)	0.02 (0.0–0.2)	0	0.02 (0.0–0.2)
30–<35	0.57 (0.3–1.0)	0.38 (0.2–0.8)	0.05 (0.0–0.3)	0.14 (0.0–0.4)	0	0	0	0	0
35+	0.82 (0.4–1.7)	0.24 (0.1–0.9)	0.35 (0.1–1.1)	0.12 (0.0–0.8)	0	0	0	0	0.12 (0.0–0.8)
*p* value	0.38	0.52	0.06	0.62	0.40	0.76	0.69	0.28	0.28

^a^1 traumatic ACL injury and 1 unknown reason for revision

**Fig. 2:**
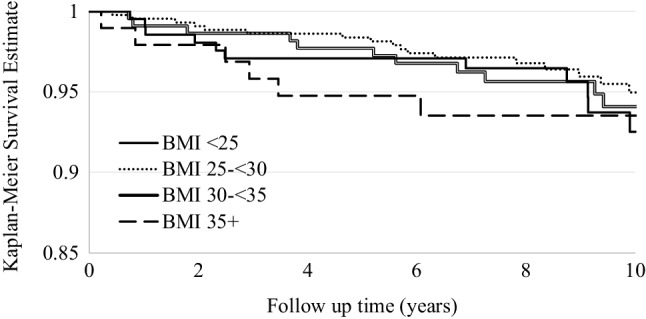
Kaplan–Meier implant survival estimate by BMI category

The cumulative 10-year survival rate of UKAs for those with a BMI < 25 was 92% (95% CI 86–96), between 25 and < 30 was 95% (CI 92–97), between 30 and < 35 was 94% (CI 90–98) and > 35 was 93% (CI 87–99) (Fig. 2). There was no significant difference between implant survival rates across the groups.

## Discussion

The most important finding of this paper is that there is no significant difference in the 10-year survivorship of implants in the different BMI groups and, in particular, no trend towards decreasing survival with increasing BMI. All groups of BMI experienced 10-year survival rates of 92% or better, highlighting good implant survival of the Oxford UKA despite BMI. Interestingly, overweight and obese class I patients experienced the highest rates of survivorship at 10 years (95 and 94.1% respectively) with patients within a healthy BMI range suffering the worst survivorship, though this was not significant. This is counter-intuitive, as most people tend to associate healthy weight range with increased implant survival. It has been argued that this is potentially due to lower levels of postoperative activity in obese patients; however, the Tegner activity scores show there is no clinically important observable difference in activity levels between BMI groups. Therefore, the similar survivorship may be due to the mobile bearing of the Oxford knee, which minimises polyethylene wear and decreases sheer stress at the bone implant interface, minimising the risk of loosening [12].

The question has also been raised that, considering obese patients are more likely to develop OA at a younger age, is it more likely that the reason for UKA revision is due to disease progression with development of lateral compartment OA? Disease progression was the most common cause in the cohort for implant revision, accounting for 50% of all revisions. Despite this, there was no significant difference in the indications for revision between different BMI groups; however, a larger sample size may be necessary to make meaningful conclusions.

Preoperative functional scores were lower in those with higher BMI, and these patients were also younger. This may reflect a relative reluctance in offering surgery to young patients compared to older patients who are more likely to be normal weight. Whilst statistically significant differences existed between BMI categories with postoperative scores, there were no consistent trends with increasing BMI and the differences between postoperative scores in the BMI categories were relatively small. When interpreting OKS data, it is essential to consider both absolute follow-up scores as well as change from the preoperative scores [22]. All groups achieved good 10-year scores, and the obese class II had the greatest improvement in score, presumably because they started off worst.

There has been a lot of attention directed to the application and use of UKAs and TKAs, with some clinical commissioning groups in the UK rationing knee arthroplasty based on BMI, with a BMI of 35 being the commonest mandatory threshold above which arthroplasty should not be undertaken [29]. It has previously been shown that BMI has no significant effect on the cost-effectiveness of knee arthroplasty [9]. Obese class II patients, with BMI > 35, have the greatest improvement in OKS, and their 10-year OKS is better than obese patients with BMI 30–35. Additionally, with comparable implant survival in all BMI subgroups, rationing should not be based on BMI and, in particular, a threshold of 35 is not justified. Obesity is associated with multiple health complaints [2], and encouragement of weight reduction should be supported but not at the expense of delaying knee arthroplasty.

There are few long-term studies that have assessed the impact of BMI on implant survivorship and postoperative functional outcomes of mobile-bearing UKAs. Our study is supported by the findings of Murray et al. [23], who showed that in 2438 patients with a mean follow-up of 5 years, survival rate of the mobile bearing UKA does not decrease with increasing BMI and that obese patients had the greatest improvement in functional scores. Using the same cohort of patients at shorter follow-up, Pandit et al. [27] reported that there was no difference in clinical outcomes in patients weighing greater or less than 82 kg at 5-year follow-up and Kuipers et al. [18] who showed no reduction in implant survival at a mean follow-up of 2.6 years. Obesity is considered by some authors to be a contraindication in fixed-bearing unicompartmental knee arthroplasties due to concern over increased polyethylene wear and implant loosening [11]. Despite this, several authors have undertaken fixed-bearing UKA in high BMI patients and have reported variable findings: Cavaignac et al. [6] in 212 UKA with mean follow-up of 12 years, reported 10-year survival of 94% in those with BMI < 30, and 92% in those > 30. Woo et al. [33] found preoperative BMI did not influence implant survivorship at mean follow-up of 5.4 years in 673 UKA. Tabor et al. [30] reported greater survivorship in obese patients with follow-up to 20 years. Conversely, in a cohort of 67 knees with a mean follow-up of 3 years, Bonutti et al. [5] reported a higher risk of implant failure with a low survival rate of 88% in patients with a BMI > 35 kg/m^2^, compared to a survival rate of 100% in patients with a BMI < 35 kg/m^2^. Berend et al. [4] similarly found an early implant failure rate of 22% in patients weighing greater than 32 kg/m^2^ at a mean follow-up of 40.2 months.

This paper has limitations. There are many reasons why rationing may take place, and our paper focusses on the most important which are implant survival and patient function. Patient selection is important in the successful outcome of UKA. The senior authors offer medial UKA to any patient who is considered to benefit from knee arthroplasty and satisfies the recommended indications. This is regardless of patient BMI. Other surgeons may use different indications. In addition, there is no treatment comparator group. This study compared the results of different BMI groupings within UKA and demonstrated that there are similar outcomes across groups. UKA can be used in up to 50% of patients requiring knee arthroplasty, but this study was unable to make direct comparisons to TKA within different BMI groupings. However, large matched studies have shown numerous benefits to UKA, and this study has shown that the outcomes are consistent across BMI groupings. This study cannot comment on extremes of BMI range. There were not a large number of obese class III (BMI > 40 kg/m^2^) patients in our cohort, with only 27 patients in this class. Finally, all patients in this study were operated on by two designer surgeons who have a high surgical volume of UKA. This brings into question the generalisability of this paper as previous studies have indicated higher failure rates in hospitals with low surgical volumes of UKAs [3]. It has, however, been shown that if surgeons adhere to the recommended indications, they can expect to achieve optimum result with a knee arthroplasty practice where surgeons are performing at least 20% of their knee arthroplasties as UKA [15].

This paper benefited from being a prospective study with long-term follow-up of a large cohort; therefore, it provides an accurate picture of the impact of BMI on the long-term outcome of medial Oxford UKA. It provides further evidence that high BMI does not lead to inferior patient reported or survival outcomes, and supports the recommendation that a BMI threshold should not be considered a contraindication with respect to these outcomes.

## Conclusion

This study found no difference in implant survival or indications for revision amongst patients with higher BMIs. Additionally, the most obese patients, with BMI ≥ 35 (obese class II), experience the greatest improvement in function following a UKA. Based on these findings, when UKA is used with appropriate indications, high BMI should not be considered to be a contraindication and a rationing threshold of 35 kg/m^2^ is unjustified.
